# Amelioration of oxidative stress-mediated apoptosis in copper oxide nanoparticles-induced liver injury in rats by potent antioxidants

**DOI:** 10.1038/s41598-020-67784-y

**Published:** 2020-07-02

**Authors:** Samy A. Abdelazeim, Nagwa Ibrahim Shehata, Hanan Farouk Aly, Shams Gamal Eldin Shams

**Affiliations:** 1 Biochemistry Department, Faculty of Pharmacy, Cairo University, Cairo, Egypt; 2 Therapeutic Chemistry Department, National Research Center, Dokki, Giza, Egypt

**Keywords:** Biochemistry, Molecular biology

## Abstract

The purpose of this study is to investigate the therapeutic efficacy of individual or combined doses of dehydro-epiandrosterone (DHEA) and quercetin in ameliorating some biochemical indices in liver of CuO-NPs intoxicated-rats. CuO-NPs (50 nm) was administered as a daily oral dose 100 mg/kg for 2 weeks to rats followed by the fore-mentioned antioxidants for 1 month. We highlighted the therapeutic effect of DHEA and quercetin against CuO-NPs toxicity through monitoring the alteration of liver enzyme activity, antioxidant defense mechanism, necrosis, apoptosis, histopathological alterations, and DNA damage. The rats given CuO-NPs only showed marked significant elevation in liver enzymes, alteration in oxidant-antioxidant balance and an elevation in the hepatic inflammatory marker; tumor necrosis factor-α. Additionally, over expression of both caspase-3 and Bax proteins were detected. Whereas, Bcl2 was down regulated and DNA fragmentation was elevated. Moreover, Histopathological examination of hepatic tissue reinforced the previous biochemical results. Co-treatment with either DHEA, quercetin alone or in combination ameliorated the deviated parameters with variable degrees against CuO-NPs toxicity in rat. In conclusion, our findings suggested that the aforementioned treatments exert therapeutic effect in CuO-NPs toxicity by diminishing oxidative stress, mRNA gene expression and hepatic tissues DNA damage.

## Introduction

Nanotechnology is now involved widely in human lives with a lot of applications, especially in medicine, biological sciences, diagnosis, drug delivery, food industry, paints, electronics, sports, environmental cleanup, cosmetics and sunscreens^1^.

Copper oxide nanoparticles (CuO-NPs) were among the first engineered nanoparticles, due to their exclusive properties and essential applications in magnetic, thermal, electrical and sensor devices as well as cosmetics. This makes human beings exposed to CuO-NPs and their potential adverse effects^2^. Biodistribution experiments revealed that liver, kidney and spleen are the target organs for engineered nanoparticles after uptake by the gastrointestinal tract^3^.

Due to their minute size and surface properties, metal oxide NPs may cross biological barriers and accumulate in different organs^4^. It has been stated that liver is one of the most targeted organs for NPs after they enter the body through any route^5^. Several studies have shown that NPs have cytotoxic effect in liver cells^5,6^. However, the primary mechanism of apoptosis in liver cells due to CuO-NPs exposure is largely deficient.

The key process in cancer development and progression is apoptotic cell death^7^. The ratio of Bax/Bcl-2 represents a cell death switch, leading to apoptosis^8^. Bcl-2 protein has an anti-apoptotic effect, whereas Bax is known for its pro-apoptotic effect^9^. Moreover, apoptotic stimuli leading to destabilization of the mitochondrial integrity precedes activation of caspases which play a central role in the execution of apoptosis^10^.

It has been reported that there is a direct link between oxidative stress, genotoxicity, and apoptosis^11^. Oxidative stress plays an important role in the mechanism of toxicity of many compounds either by the production of reactive oxygen species (ROS) or by depletion of the cellular antioxidant capacity. Oxidative stress also, affects cellular integrity when the generation of ROS exceeds the antioxidant defense mechanism^12^. There are many evidences showing that NPs amplify ROS generation that were the main cause of cell death in several types of cultured cells^13,14^.

The human body is constantly attacked by chemicals that may cause DNA damage by non-oxidative and oxidative mechanisms, which can lead to carcinogenesis. For detecting and analyzing DNA damage and repair at a single cell level in a variety of organs and cells of mammals, the comet assay is used which is a simple, rapid and sensitive gel electrophoresis technique^15^. The comet assay is widely used in monitoring and assessment of genotoxicity as, it allows any viable eukaryotic cell to be analyzed for DNA damage^16^. Moreover, the comet assay can be applied in other studies including DNA repair, environmental and human biomonitoring as well as clinical studies. Computable study for DNA damage by comet assay has generated several parameters, including Tailed Nuclei, Tail Length, % DNA in the Tail, and Tail Moment^17^.

Dehyroepiandrosterone (DHEA) is a naturally occurring adrenal steroid in mammals synthesized from cholesterol and metabolized to androstenedione and estrogens. The decrease in its production is considered as the most characteristic age-related change in the adrenal cortex^18^. DHEA has various actions, such as anti-obesity, anti-diabetic and anti-carcinogenic effects when administered to mice and rats^19^. However, DHEA administration effects can be antioxidant^20^ or pro-oxidant^21^, depending on the administered dose and specific tissue^22^. It was also reported that DHEA has a dose-dependent protective effect against oxidative stress-induced endothelial dysfunction in ovarictomized rats^23^.

Quercetin (3,5,7,30,40-pentahydroxyflavone) is considered as one of the most widely distributed flavonoids, present in fruits, vegetables, and many other dietary sources^24^. This compound has been reported to have anti-atherogenic, anti-inflammatory, anti-histaminic and anti-hypertensive properties responsible for its beneficial effects against cardiovascular diseases^25,26^. Quercetin is an antioxidant which scavenges superoxide in ischemia–reperfusion injury^27^, protects against oxidative stress induced by ultraviolet light^28^ and inhibits angiogenesis and carcinogenesis^29,30^. Moreover, quercetin has been shown to regulate the functions of hepatic stellate and Kupffer cells^31^. Additionally, quercetin in combination with arginine can ameliorate nano-zinc oxide nephrotoxicity in rats^32^.

The discovery of novel therapeutic agents against NPs' toxicity remains a challenge. Therefore, the present study was carried out to investigate the alteration in hepatic and serum biochemical parameters and histopathological alterations induced by CuO nanoparticles in male rats and a trial to ameliorate their harmful effects by using DHEA, quercetin either alone or in combination which are well established as antioxidants to alter the oxidative damage, hepatotoxic effects of CuO-NPs. Histopathological investigation was carried out to confirm the biochemical results.

## Materials and methods

### Chemicals

CuO NPs (particle size 50 nm), DHEA and quercetin were purchased from Sigma-Aldrich Co (St. Louis, MO, USA). All other chemicals were of the highest analytical grade available.

### Animals

The animals utilized in this study were 50 adult male albino rats of Wistar strain, weighing 120–125 g, supplied by the animal house of National Research Center (Dokki, Giza, Egypt). Animals were kept for 2 weeks to accommodate to laboratory conditions and were allowed free access to standard diet and water all over the period of the experiment. The standard diet was supplied by El-Nasr pharmaceutical company. It was composed of 72.2% carbohydrates, 3.4% fats, 19.8% proteins, 3.6% cellulose, 0.5% vitamins &minerals and 0.5% salts. Notably, all animals were fasted for 3 h prior to CuO NPs administration.

### Ethics

Anesthetic procedures and handling of animals were complied with the ethical guidelines of the Medical Ethical Committee of the National Research Centre, Cairo, Egypt with an ethical approval number (Approval no: 10131) and the Ethics committee of the Faculty of Pharmacy, Cairo University. All precautions were taken for being sure that the animals not suffer at any stage of the experiment.

### Experimental design

Rats were randomly divided into five groups, each of 10 rats according to the following schedule:

- *Group 1* Animals received saline intraperitoneally (i.p.) and served as normal control group.
- *Groups 2–5* The experimental model of CuO-NPs intoxication was achieved by giving animals a daily i.p. dose of 100 mg/kg body weight CuO-NPs suspension for 2 weeks^2^.
- *Group 2* The CuO-NPs intoxicated Animals were left untreated till the end of the experiment.
- *Group 3* The CuO-NPs intoxicated Animals were treated with daily oral dose of DHEA 300 mg/kg body weight^33^.
- *Group 4* The CuO-NPs intoxicated Animals were treated with daily i.p. quercetin 150 µg/kg body weight^34^
- *Group 5* The CuO-NPs intoxicated Animals were treated with daily oral dose of DHEA 300 mg/kg body weight and an i.p. dose of quercetin (150 µg/kg body weight/day).

Treatment was carried throughout a period of 3 weeks month after CuO-NPs intoxication.

### Blood sampling

At the end of the experimental period, rats were fasted overnight and slightly anesthetized with diethyl ether and blood samples were collected from the sublingual vein. Sera were separated by centrifugation at 4,000 rpm for 10 min and were kept at − 80 °C for subsequent estimation of alanine and aspartate aminotransferase activities, total bilirubin, albumin, tumor necrosis factor alpha (TNF-α), hepatocyte growth factor (HGF), caspase-3 activity, Bax and Bcl-2 m-RNA levels according to the instructions provided by the manufacturer of the kits.

### Preparation of liver homogenate

Animals were sacrificed by cervical dislocation and liver tissues were separated, blotted dry, and then weighed and were divided into three portions. The first portion was homogenized in PBS (phosphate buffered saline) solution, pH 7.4, containing 0.16 mg/ml heparin to remove any red blood cells and clots then, centrifuged at 4,000 rpm for 15 min at 4 °C. The supernatant was removed for the assay of MDA, GSH, NOx and catalase. The second portion was used for determination of comet assay and the third portion was embedded in 10% formaldehyde, for subsequent histopathological examinations^35^.

### Measured parameters

#### Serum levels of aminotransferases, albumin and total bilirubin

AST and ALT were determined in serum by the method of Reitman and Frankel^36^, total bilirubin was measured in serum according to the method of Walter and Gerade^37^ and albumin level according to the method of Doumas et al.^38^ Colorimetrically using Biodiagnostic kits (Egypt) following the manufacturer’s instructions.

#### Determination of serum caspase-3 activity

Serum caspase-3 activity was measured by a quantitative ELISA technique using the kit provided by R and D systems (MN, USA) according to the manufacturer’s instructions^39^.

#### Quantitative reverse-transcription polymerase chain reaction (qRT-PCR) for analysis of serum Bax and Bcl2 mRNA levels

Total RNA was extracted from serum using RNeasymini kit (Qiagen, CA, USA). The isolated RNA was quantified using UV spectrophotometer (Beckmn, USA) and the purity of RNA was verified with 260/280 nm ratio ranging from 1.9–2.1. The integrity of RNA was assessed by gel electrophoresis. The total RNA (0.5–2 μg) was used for cDNA conversion using high capacity cDNA reverse transcription kit (Fermentas, USA). Real-time qPCR amplification and analysis were performed using an Applied Biosystem with software version 3.1 (StepOne, USA). The primer sequences were provided by Shine Gene, China.

The PCR reactions included 10 min at 95 °C (activation), followed by 40 cycles at 94 °C for 15 s (denaturation) and 60 °C for 1 min (annealing/extension)^40^. The expression level was calculated from the PCR cycle number (C_T_) where the increased fluorescence curve passes across a threshold value. The relative expression of target genes was obtained using comparative C_T_ (ΔΔC_T_) method. The ΔC_T_ was calculated by subtracting β-actin C_T_ from that of the target gene whereas ΔΔC_T_ was obtained by subtracting the ΔC_T_ of the calibrator from that of the test sample. The relative expression was calculated from 2^−ΔΔCT^ formula based on the method of Pfaffl^41^.

#### Hepatic CAT activity

CAT activity (U/g) was assayed in liver homogenate according to the method of Aebi^42^ using Biodiagnostic kit (Egypt). CAT reacts with a known quantity of H_2_O_2_. The reaction is stopped after exactly one min. with a catalase inhibitor. In the presence of horseradish peroxidase (HRP), the remaining H_2_O_2_ reacts with 3,5-dichloro-2-hydroxybenzene sulfonic acid (DHBS) and 4-aminophenazone (AAP) to form a chromophore with color intensity inversely proportional to the amount of CAT in the original sample.

#### Hepatic GSH level

Hepatic GSH (mg/g tissue) was assayed in liver homogenate according to the method of Moron et al.^43^ using Biodiagnostic kits (Egypt) following the manufacturer’s instructions. The method is based on the development of a relatively stable yellow colour when 5,5-dithiobis-2-nitrobenzoic acid (DTNB) is added to sulfhydryl compounds.

#### Hepatic MDA level

MDA (nmoL/g tissue) was determined in liver homogenate by a colorimetric assay according to the method of Satoh^44^, using Biodiagnostic kit (Egypt). Thiobarbituric acid (TBA) reacts with MDA in acidic medium at 95 °C for 30 min to form thiobarbituric acid reactive product. The absorbance of the resultant pink product was measured at 534 nm.

#### Hepatic nitrate/nitrite (NOx) level

Nitric oxide was determined in liver homogenate by the colorimetric assay method of Montgomery et al.^45^ using Biodiagnostic kits (Egypt), following the manufacturer’s instructions. In acid medium and in the presence of nitrite, the formed nitrous acid is diazotized with sulphanilamide and the product is coupled with N-(1-naphtyl) ethylenediamine. The resulting azo dye has a bright reddish-purple color which can be measured at 540 nm.

#### Hepatic percent of DNA damage by comet assay

Single cell gel electrophoresis assay (also known as comet assay) was performed as previously described by Singh et al.^4^ Comet assay is a quick, accurate and simple method for detecting DNA damage. In this method, cellular DNA was detected when electric field was used that leads to the migration of DNA fragments from the cell nucleus through an agarose gel using fluorescent dyes, resulting in a comet-like shape. All breaks of DNA migrate freely into the tail of the comet. The tail length and the percentage of total DNA in the tail is directly proportional to DNA damage, which is also, related to the frequency of breaks. Dimmed light was used in all the steps of comet assay, to prevent additional DNA damage. A Leitz Orthoplane Pi fluorescence microscope (magnification 200) equipped with an excitation filter of 515–560 nm and a barrier filter of 590 nm was used for Image analysis. The microscope was connected through a camera to a computer-based image analysis system (Comet Assay IV software, Perspective Instruments). One hundred randomly selected cells per slide were scored.

#### Histopathological investigations

Paraffin-embedded tissue sections (5 µm) of liver were cut on a slidge microtome (Leica RM2135 Rotary Microtome, Wichita, KS, USA) and stained with haematoxylin and eosin (H &E) stain for subsequent histopathological examination by the light microscope^46^.

### Statistical analysis

Data were expressed as means ± SEM. The results were statistically analyzed by one-way analysis of variance (ANOVA) using SPSS (Statistical Package for the Social Sciences, version 16.0.1, Chicago, IL) software. Individual treatment means were compared post hoc by the Scheffé test. Differences were considered significant at *P* < 0.05.

## Results

### Effect of CuO-NPs and other treatments on rat serum levels of liver biomarker

As shown in Table 1, CuO-NPs induced hepatotoxicity as reflected by elevated levels of serum ALT, AST, and bilirubin; whereas serum total protein is reduced (*P* < 0.05) compared to normal control values. The activity of ALT was more affected, than that of AST activity. On the other hand, Co-administration of either (DHEA) or quercetin (Q) had significantly increased serum total protein level (*P* < 0.05) and lower serum ALT, AST and bilirubin levels compared with CuO-NPs induced hepatotoxicity group (*P* < 0.05). However, Co-administration with (CuO-NPs + DHEA + Quercetin) restored the changes in liver biomarkers levels back to near the normal control values. Therefore, only, the Co-administration with (CuO-NPs + DHEA + Quercetin) group offered a protective effect against hepatotoxicity induced by CuO-NPs.

**Table 1:** Effect of dehydro-epiandrosterone (DHEA), quercetin and their combination on serum aminotransferases activities, serum albumin and total bilirubin levels in nano-copper oxide (CuO NPs) intoxicated rats.

Groups	Parameters			
	Serum ALT (U/ml)	Serum AST (U/ml)	Serum albumin (g/dl)	Serum total bilirubin (mg/dl)
Control	21.7 ± 0.63	19.3 ± 0.49	5.89 ± 0.09	0.68 ± 0.03
CuO NPs	99.3 ± 0.57a	156 ± 1.34a	1.65 ± 0.03a	4.85 ± 0.07a
CuO NPs + DHEA	71.2 ± 1.18 ab	43.5 ± 0.47ab	2.96 ± 0.05ab	4.06 ± 0.04ab
CuO NPs + quercetin	56.7 ± 0.85ab	40.8 ± 0.66 ab	2.42 ± 0.05ab	1.26 ± 0.04ab
CuO NPs + DHEA + quercetin	44.8 ± 1.91ab	22.8 ± 0.66b	4.68 ± 0.22ab	1.02 ± 0.02ab

Values are expressed as mean ± SEM (n = 10). *P* value < 0.05 is considered significant. (a) Significantly different from normal control group. (b) Significantly different from nano-copper oxide intoxicated rats group.

### Effect of CuO-NPs and other treatments on rat liver oxidative stress

**Figure 1:**
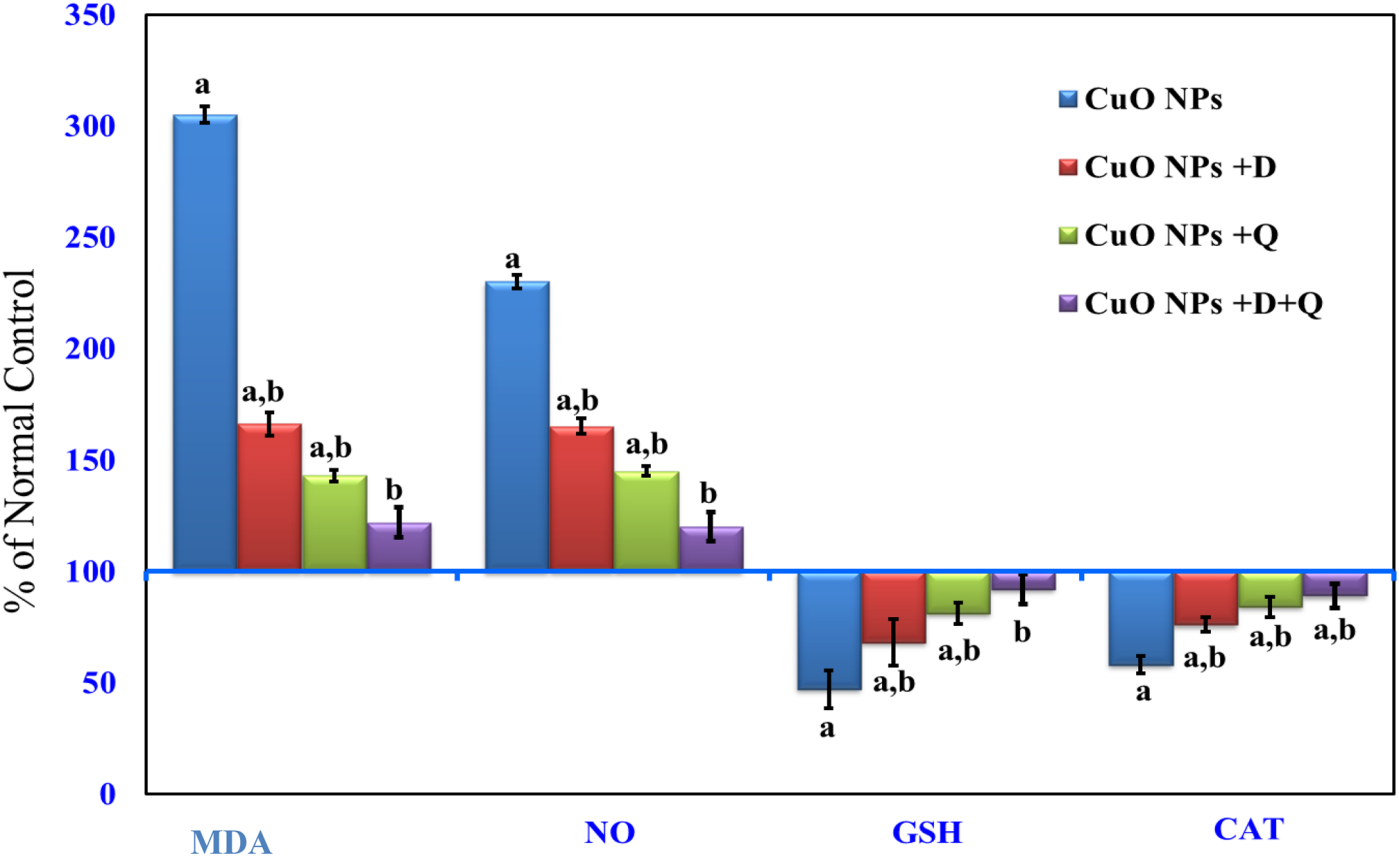
Effect of DHEA, quercetin and their combination on hepatic levels of MDA, nitrite/nitrate (NOx), GSH and catalase in CuO-NPs intoxicated-rat. Values are expressed as % of normal control ± % SEM, (n = 10). *P* value < 0.05 is considered significant. (**a**) Significantly different from normal control group. (**b**) Significantly different from nano-copper oxide intoxicated rats group.
- CAT and GSH:
  Administration of CuO-NPs to rats resulted in a significant reduction (*P* < 0.05) in liver GSH level and CAT activity compared to normal control rats (Fig. 1). Both DHEA and quercetin co-treatment showed a marked elevation in GSH level and antioxidant enzyme activity compared to CuO-NPs intoxicated-rats. In contrast, Co-administration of the (CuO-NPs + DHEA + Quercetin) resulted in a normalization of the GSH level and CAT activity as compared to CuO-NPs intoxicated-rats alone (Fig. 1). Interestingly, the result supports and confirms the anti-lipid peroxidase as well as effective antioxidant properties of (DHEA + Quercetin) against the damaging effects of free radicals produced by CuO-NPs.

- Lipid peroxidation:
  As indicated in Fig. 1, hepatic levels of MDA and NO were significantly (*P* < 0.05) increased in CuO-NPs intoxicated-rat as compared to control rats. Concomitant treatment with DHEA, quercetin either alone or in combination resulted in a significant decrease in hepatic levels of MDA and NO compared to CuO-NPs intoxicated-rat alone.

### Effect of DHEA, quercetin and their combination on levels of TNF-α and HGF

**Figure 2:**
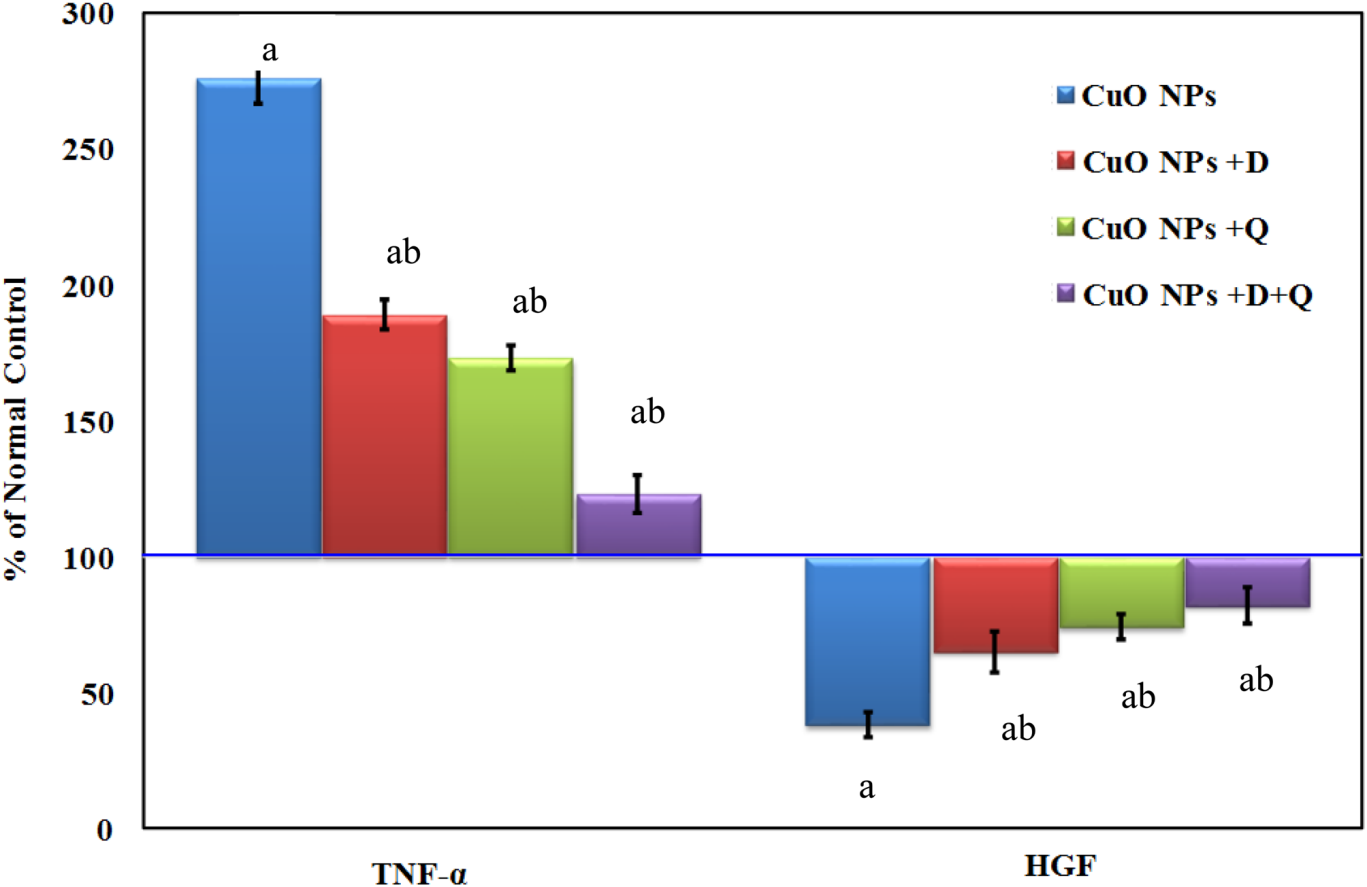
Effect of DHEA, quercetin and their combination on serum tumor necrosis factor-alpha (TNF-α) and hepatocyte growth factor (HGF) levels in CuO NPs intoxicated-rat. Values are expressed as of normal control ± %SEM, (n = 10). *P* value < 0.05 is considered significant. (a) Significantly different from normal control group. (b) Significantly different from nano-copper oxide intoxicated rats group.

As shown in Fig. 2, the serum levels of TNF-α was significantly increased associated with marked decrease of hepatocyte growth Factor (HGF) levels in CuO-NPs intoxicated-rat, reaching 469 ± 2.73 (286.7) and 15.9 ± 1.18 (23.6%), respectively, as compared to the normal control. Administration of DHEA or quercetin reduced significantly the levels of TNF-α accompanied with significant elevation of (HGF) levels compared to CuO-NPs intoxicated group. Meanwhile, Co-administration of the (CuO-NPs + DHEA + Quercetin) resulted in returning back the TNF-α and (HGF) levels to near the normal control values (Fig. 2).

### Effect of DHEA, quercetin either alone or in combination on apoptosis

**Figure 3:**
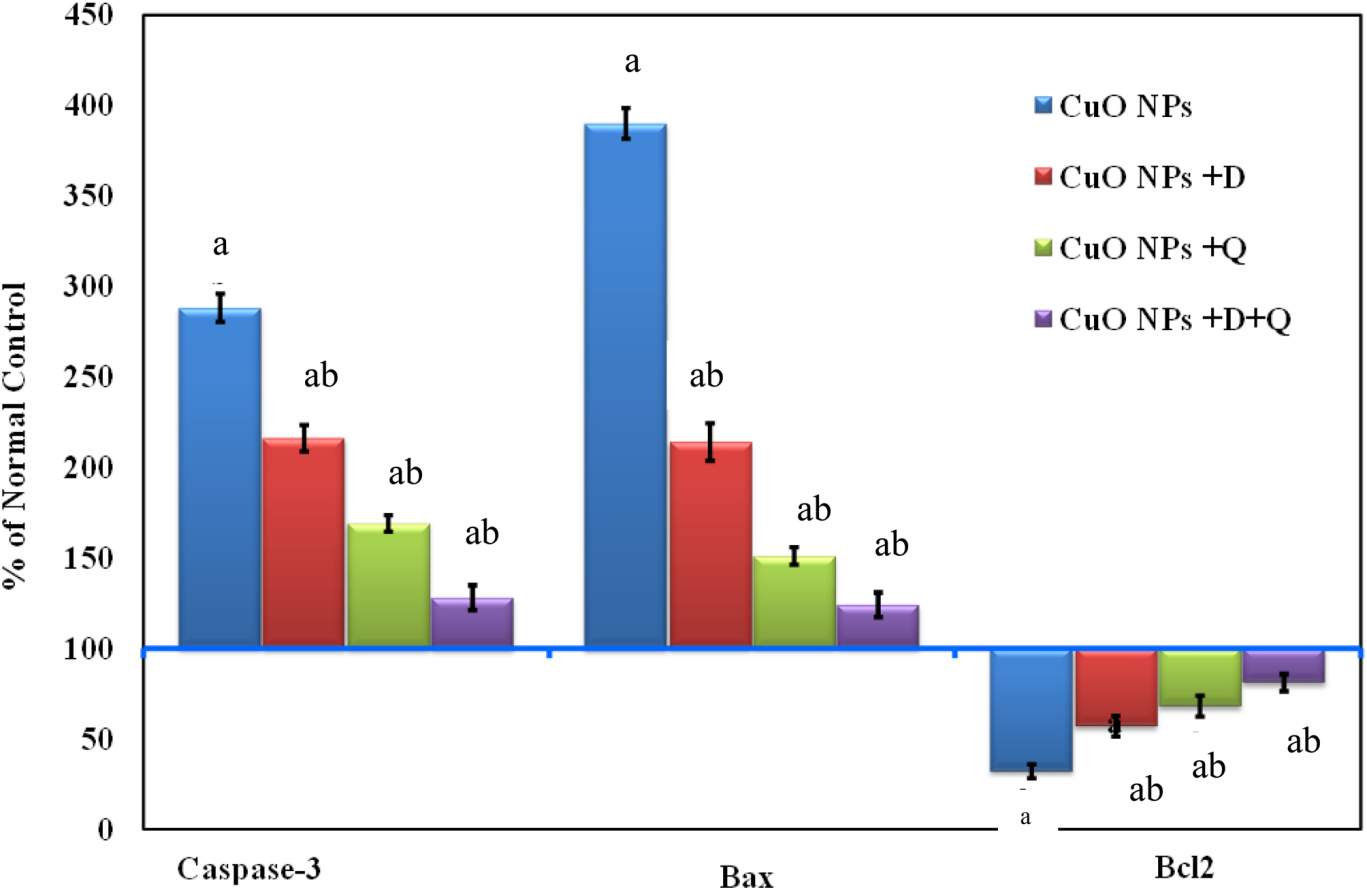
Effect of DHEA, quercetin and their combination on serum caspase-3 activity and mRNA levels of Bax and Bcl2 following CuO NPs intoxicated rats. Values are expressed as % of normal control ± % SEM (n = 10). *P* value < 0.05 is considered significant. (a) Significantly different from normal control group. (b) Significantly different from nano-copper oxide intoxicated rats group.

The data in Fig. 3 indicated that CuO-NPs intoxication caused a significant elevation of serum caspase-3 activity and up-regulation in mRNA level of Bax by almost (threefold and fourfold respectively) associated with a significant down-regulation mRNA level of Bcl2 as compared to the control value. Administration of DHEA or quercetin reduced significantly the levels of serum caspase-3 activity and mRNA level of Bax accompanied with significant elevation of Bcl2 levels compared to CuO-NPs intoxicated group. Besides, the combination regimen considerably reversed the level of these parameters back near to the normal value.

Meanwhile, these data suggested that the pre-administration of DHEA and quercetin were able to restrict the pro-apoptotic signals (Caspase-3 and Bax) and enhance the anti-apoptotic signal (Bcl2), thus offering protection.

### Effect of DHEA, quercetin either alone or in combination on DNA damage

**Figure 4:**
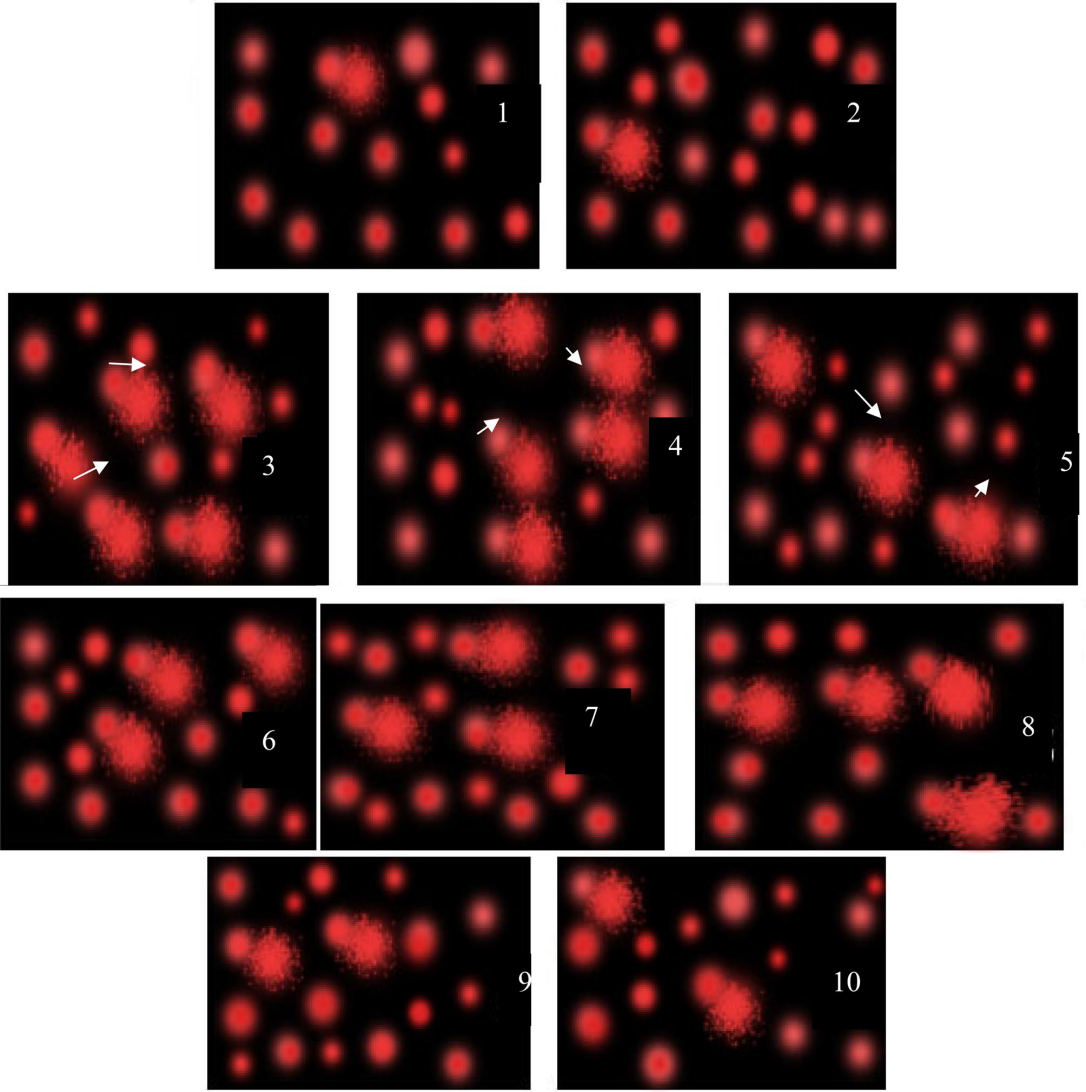
Effect of DHEA, quercetin and their combination on the percentage of DNA damage by comet assay in the liver tissue of CuO NPs-intoxicated rats. **Slides (1,2):** The control group with no significant DNA damage. **Slides (3,4):** CuO NPs intoxicated group with a remarkable percent of DNA damage. **Slides (5,6)** and **(7,8)** are of the CuO NPs + DHEA and CuO NPs + quercetin, respectively with lesser percent of DNA damage relative to CuO NPs group. **Slides (9,10):** the CuO NPs intoxicated group treated with the combined therapy of DHEA and quercetin, showing the most significant reduction in the percent of DNA damage.

The data in Table 2 revealed that CuO NPs intoxication produced a significant elevation in tail moment reaching 12.76 ± 0.61( 570%)of the control group. On the other hand, administration of either DHEA or quercetin significantly reduced tail moment as compared to CuO NPs-intoxicated group reaching 9.47 ± 0.41 (423%) and 7.85 ± 0.18 (350%) of the control group, respectively. It is obvious that the combination regimen produced the most marked reduction in tail moment as compared to CuO NPs-intoxicated group reaching 7.72 ± 0.23 (345%) of the control group. As indicated in Fig. 4, the control group showed no significant DNA damage while CuO NPs intoxicated group showed a remarkable percent of DNA damage. Administration of either DHEA or quercetin showed respectively lesser percent of DNA damage relative to CuO NPs group. It was obvious that the CuO NPs intoxicated group treated with the combined therapy of DHEA and quercetin, showed the most significant reduction in the percent of DNA damage.

**Table 2:** Effect of DHEA, quercetin and their combination on the percentage of DNA damage in the liver tissues of CuO NPs-intoxicated rats.

Groups	Parameters				
	Tailed cell (%)	Untailed (%)	Tail length (µm)	DNA tail (%)	Tail moment (unit)
Control	3.66 ± 0.33	96.3 ± 0.33	1.53 ± 0.04	1.46 ± 0.04	2.24 ± 0.003
CuO NPs	17.6 ± 0.66a	82.3 ± 0.66a	3.57 ± 0.05a	3.57 ± 0.12a	12.76 ± 0.61a
CuO NPs + DHEA	13.3 ± 0.33ab	86.7 ± 0.33ab	3.11 ± 0.06ab	3.04 ± 0.07ab	9.47 ± 0.41ab
CuO NPs + quercetin	12.3 ± 0.33ab	87.6 ± 0.33ab	2.84 ± 0.04ab	2.76 ± 0.03ab	7.85 ± 0.18ab
CuO NPs + DHEA + quercetin	10 ± 0.00ab	90 ± 0.00ab	2.82 ± 0.05ab	2.73 ± 0.03ab	7.72 ± 0.23ab

Values are expressed as % of total counts in each assay. Each parameter was done in triplicate. Tail moment (unit) = tail length x% tail DNA. Data are expressed as mean ± SEM. *P *value ˂ 0.05 is considered significant. (a) Significantly different from normal control group. (b) Significantly different from nano-copper oxide intoxicated rats group.

### Histopathological findings

**Figure 5:**
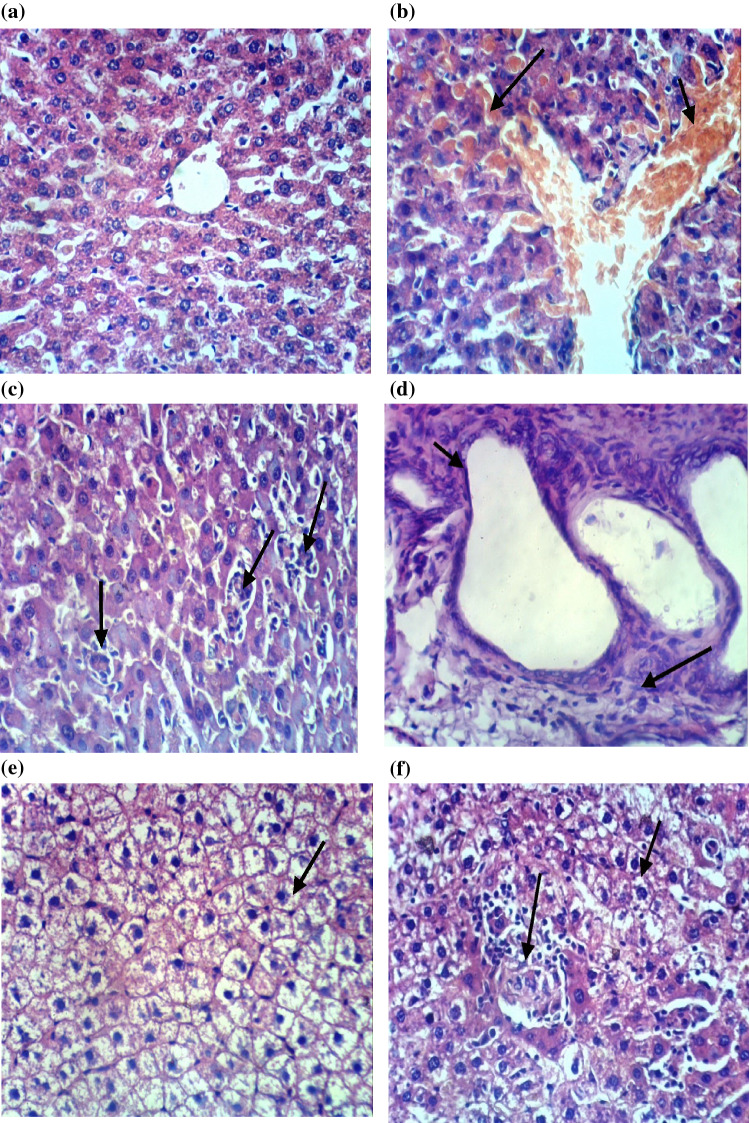

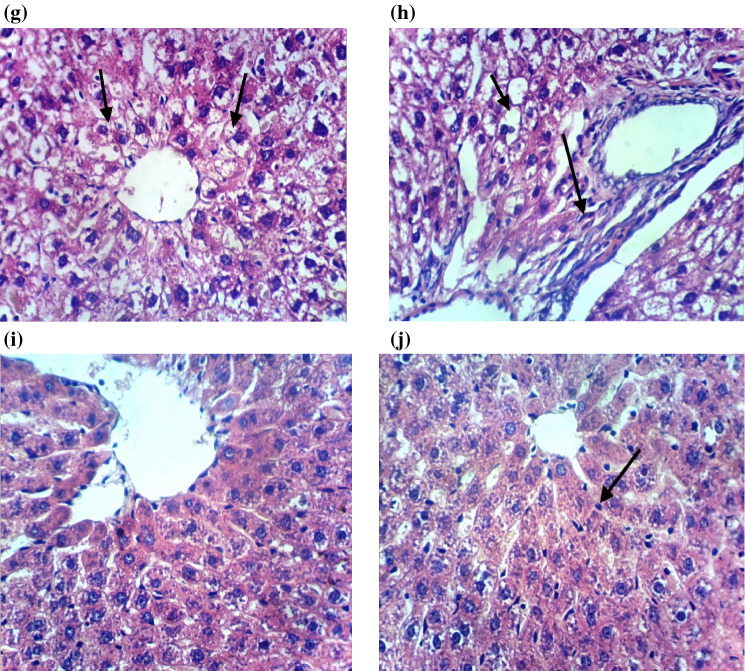
(**a**) Liver of normal control rat showing the normal histological structure of hepatic lobule (H and E × 400). (**b**) Liver of rat from CuO NPs intoxicated group showing congestion of central vein and hepatic sinusoids (H and E × 400). (**c**) Liver of rat from CuO NPs intoxicated group showing focal hepatic necrosis associated with inflammatory cells infiltration (H and E × 400). (**d**) Liver of rat from CuO NPs intoxicated group showing cystic dilatation of bile duct and fibroplasia in the portal triad (H and E × 400). (**e**) Liver of rat from CuO NPs + DHEA group showing hydropic degeneration of hepatocytes (H and E × 400). (**f**) Liver of rat from CuO NPs + DHEA group showing hydropic degeneration of hepatocytes and focal hepatic necrosis associated with inflammatory cells infiltration (H and E × 400). (**g**) Liver of rat from CuO NPs + Quercetin group showing hydropic degeneration of hepatocytes (H and E × 400). (**h**) Liver of rat from CuO NPs + Quercetin group showing hydropic degeneration of hepatocytes and few fibroblasts proliferation in the portal triad (H and E × 400). **(i**) Liver of rat from CuO NPs + DHEA + Quercetin group showing no histopathological changes (H and E × 400). (**j**) Liver of rat from CuO NPs + DHEA + Quercetin group showing Kupffer cells activation (H and E × 400).

Microscopically, the livers of control rats displayed the normal histological structure of hepatic lobules as shown in (Fig. 5a). In contrast, the livers of rats from CuO NPs-intoxicated group showed congestion of central vein and hepatic sinusoids (Fig. 5b), focal hepatic necrosis associated with inflammatory cells infiltration (Fig. 5c), cystic dilatation of bile duct and fibroplasia in the portal triad (Fig. 5d). However, the livers of rats from CuO NPs + DHEA-treated group revealed hydropic degeneration of hepatocytes (Fig. 5e) and focal hepatic necrosis associated with inflammatory cells infiltration (Fig. 5f). The livers of rats from CuO NPs + quercetin-treated group revealed no changes except for hydropic degeneration of hepatocytes (Fig. 5g) and few fibroblasts proliferation in the portal triad (Fig. 5h). Meanwhile, the livers of rats receiving the combination regimen of DHEA and quercetin showed apparently normal liver architecture with no histopathological changes (Fig. 5i) except for Kupffer cells activation in some examined sections (Fig. 5j).

## Discussion

The current study revealed that injection of CuO-NPs to rats elevated serum levels of ALT, AST, total bilirubin associated with reduced serum levels of albumin compared to control group, which suggested the presence of marked liver damage (hepatotoxicity), that was further confirmed by hepatic degeneration and necrosis appeared in histopathological examination. Previously, Mohammad Yari et al.^47^ demonstrated that CuO-NPs increased the ratio of ALT/AST and total bilirubin in serum. Elevated levels of ALT/AST ratio in CuO-NPs group indicated cellular leakage. In other study, El-Magd^48^ reported that oral administration of CuO-NPs to rats induced marked reduction in serum albumin level indicating liver damage and loss of function, suggesting the occurrence of hepatic insufficiency.

Treatment with quercetin, DHEA and their combination exhibited a significant reduction in liver enzyme activities, total bilirubin as well as a significant elevation in albumin levels as compared to CuO-NPs intoxicated group. On the other hand, the improvement in hepatic functions following the administration of these antioxidants implied their possible protective effect on hepatocytes. In harmony with our findings, Arafa et al.^49^ declared that quercetin induced a significant reduction in liver enzymes and bilirubin level in cirrhotic rats induced by carbon tetrachloride, this healing potency of quercetin may be attributed to its powerful antioxidant effect and the corrective effect of quercetin on liver markers. In agreement, Patel and Katyare^50^ declared that DHEA protect against N-dimethyl nitrosamine induced hepatocarcinoma, reduced liver enzyme activities, total bilirubin level in rats and attributed this effect due to the ability of DHEA to relieve cellular ROS, and preserve cell membrane integrity leading to prevention of enzymes leakage to the circulation, accompanied with increased cellular protein content and reduced damage to cellular DNA and enhanced protein structure^50^.

Nevertheless, the combination of the two medications ; quercetin and DHEA significantly demonstrated the most noticeable benefit of remediation on liver enzyme activities and total bilirubin level; suggesting the synergistic effect of both treatment.

NO levels and oxidative stress have been suggested to play a chief role in the mechanisms of toxicity for a number of nanoparticles including copper oxide which also, leads to activation of Kupffer cells and recruitment of inflammatory cells that mediates oxidative damage through responsive transcription factors^7, 42^. The increased hepatic NO and MDA levels go along with the inhibition of glutathione content and CAT activities by CuO-NPs observed in our study were accepted signs of oxidative stress. The reason behind the accumulation of pro-oxidants that lead to oxidative stress is the production of ROS exceeded the capacity of cellular antioxidant machineries^51,52^. Free radicals that are generated through lipid peroxidation and reactive nitrogen species that are generated when NO reacts with ROS can both lead to damage of biomolecules including DNA, proteins and lipids and leakage of liver enzymes^53,54^. Our data are in consistent with newly published findings confirming that oxidative stress is a possible mechanism implicated in CuO-NPs hepatotoxicity^51^. This indicates that CuO-NPs may exert its hepatotoxic effect through, at least in part, elevation of free radicals and decrease in intracellular antioxidant activities. Several previous studies are in consistent with our results concerning CuO-NPs toxicity in rat liver^48,51^.

On the other hand, treatment of CuO NPs-intoxicated rats with DHEA and quercetin either alone or in combination significantly reduced NO and MDA levels parallel with increased glutathione content and CAT activities which led to improvement of the encountered oxidative damage (Fig. 1). This effect could be related to the antioxidant activities of the DHEA and quercetin. The increase in glutathione and CAT activities may be beneficial in reversing the oxidative action of CuO-NPs. Our results are in line with those of Bahar et al.^55^ who used quercetin to attenuate neuroinflammation in Mn-treated rats. In tune Aly et al.^56^ used DHEA to attenuate Al-intoxicated ovariectomized rats and reported that their results may be due to the fact that DHEA is a natural steroid hormone which is a specific activator of peroxisome proliferator-activated receptor-α (PPARα)^57^. An upregulation of mRNA and protein levels of a number of peroxisomal and non-peroxisomal-associated enzymes and structural proteins, among them are the antioxidant enzymes glutathione and CAT, is caused by such activation^58^.

In the current investigation, oral administration of CuO-NPs caused a significant elevation in serum TNF-α accompanied by significant decrease in HGF levels. These findings coincide with the previous report of Carrillo-Vico et al.^59^ who declared that, activation of pro-inflammatory cytokine TNF-α in a septic shock model and in thioacetamide intoxication^60,61^. Aditionally, the elevation in serum level of TNF-α in the present study may be related to the increased ROSproduction which is associated with TNF-α dependent activation of endothelial cells^62^. Moreover, in agreement with our results, the reports of Arends et al.^63^ who found reduction in the level of HGF after H_2_O_2_-induced toxicity in bile duct epithelial cells.

The elevation in TNF-α was blocked noticeably post administration of Quercetin or DHEA or their combination*.* This was confirmed by other previous studies that have reported that quercetin has a potent inhibitory activity against the production of TNF-αin lipopolysaccharide stimulated Kupffer cells^31^. The therapeutic effect of DHEA against TNF-α induced ROS production has been observed in rabbit renal tissue and brain where it protects against oxidative damage by preventing the reduction in antioxidant enzyme activities^64,65^. Since that, HGF is the most potent stimulator of hepatocyte proliferation and exerts multiple biological properties in the liver, including mitogenic, anti-fibrotic, and cytoprotective activities^66^. Hence, it may be speculating that, the hepatocytotoxic effect is resulting from the low HGF level in intoxicated rats. However , treatment of intoxicated rats with quercetin and ∕or DHEA markedly elevated HGF level, and these results coincide with Sicard et al.^67^ who showed that DHEA caused chromaffin cell proliferation through increase the effect of growth factors suggesting, the role of treatments as mitotic and cytoprotective effects as explained by Arends et al.^62^ that showed HGF may improve viability in bile duct epithelia cells after H_2_O_2_ induced toxicity by proliferation, strengthening the intrinsic antioxidant defenses, and/or by an attenuation of apoptosis.

Evidence from this study indicated that CuO-NPs possess the genotoxic potential to interact with DNA and cause alterations in mammalian cells in vivo that was indicated by an increase in tail moment through the comet assay. The integrity of genomic DNA is constantly under threat, even in healthy cells. Endogenous ROS or errors in replication or recombination, as well as environmental toxicants can cause a great harm to cellular DNA. In general, harmful alterations in the genetic material include chromosomal aberrations and point mutations involving a change in a single base^17^. Awasthi et al.^17^ and Wang et al.^68^ proved that oxidative stress was the primary toxic effect of CuO-NPs leading to DNA damage as increased level of ROS by NPs can cause DNA point mutations and/or induce single- or double-strand breaks^4^. In addition, Cu^2+^ has been previously reported to decrease cell viability by binding to DNA resulting in cell death^51, 69^.

Our study revealed that treatment of CuO-NPs intoxicated rats with DHEA and quercetin either alone or in combination alleviate CuO-NPs induced DNA damage which was proved by the decrease of comet length and tail moment. These results are similar to those of Ding et al.^70^ who showed that pre-treatment with DHEA prevented H_2_O_2_-induced DNA damage in Leydig cells in a dose-dependent pattern and this effect was attributed to the inhibition of •OH generation. Our findings are also in accordance with a new study reporting that quercetin decreased the severity of acrylamide-induced DNA damage in the rat liver through indirect induction of detoxifying genes, which might promote detoxification of acrylamide and decrease its toxicity^71^. Quercetin antioxidant effect is due to its scavenging oxy radicals ability at several sites throughout the lipid bilayer, this ability is due to its pentahydroxyflavone structure that enables it to chelate metal ions via the orthodihydroxyphenolic arrangement^71^.

In addition, the current results at the molecular level, showed a substantially high expression of the pro-apoptotic protein (Bax) in the liver tissues of CuO-NPs intoxicated rats, while the anti-apoptotic protein; Bcl-2 was significantly down-regulated. Together, this implies that the Bax/Bcl-2 ratio, a main index for apoptosis, might be significantly elevated; indicating CuO-NPs enhanced apoptosis in the livers of intoxicated rats. Also, the present study showed a significant inhibition of serum caspase-3 activity in CuO-NPs intoxicated group. This may be attributed to the over production of ROS which induced a significant elevation of caspase­3 activity leading to apoptotic condition^72,73^.

Our findings are supported by the previous reports which showed that CuO-NPs induced apoptosis in the rat kidney and liver directly through the alteration of apoptotic gene expression^50,68^. Accumulating evidences have indicated that NPs could induce cellular apoptosis by targeting the mitochondrial apoptotic pathway^74–76^. This may be through the activation of cytochrome c release from the mitochondria, down-regulation of Bcl-2 expression, up-regulation of Bax expression, translocation of Bax into the mitochondrial membrane, and activation of caspase-3^50^. Another major oxidative stress response is the release of intracellular Ca^2+^, which leads to mitochondrial perturbation and cell death^77^. In line with our results, Ibrahim et al.^51^ reported that NPs, following endocytotic uptake, are able to induce mitochondrial damage through the direct interaction of undissolved NPs with the ROS-derived lipid peroxides causing disruption of membrane integrity and release of apoptotic enzymes^78^. This could lead to destruction of hepatocytes and activation of hepatic stellate cells, which play a central role in liver damage and fibrosis^51^.

On the other hand, our study revealed that treatment of CuO-NPs intoxicated rats with the combination of DHEA and quercetin down-regulated the expression of Bax and up-regulated Bcl-2 expression. The present results also declared significant inhibition of caspase-3 activity in groups treated with DHEA and quercetin either alone or in combination. This, in turn, provides evidence for the anti-apoptotic properties of DHEA and quercetin. These findings coincide with the previous report of Ding et al.^70^ which highlighted that DHEA prevented H_2_O_2_-induced Leydig cells apoptosis mainly through inhibiting the expression of Bax which resulted in the inhibition of caspase-9 and caspase-3 activities. The mechanism by which DHEA could stimulate Bcl-2 expression is that DHEA binds to and activates G-protein coupled membrane receptor alpha inhibitory subunit (Gαi) that, in turn, activates proto-oncogenic tyrosine kinase c (Src), protein kinase C (PKC) and MAPK/ERK pathway. These kinases activate the prosurvival transcription factors CREB which stimulate the expression of antiapoptotic proteins such as Bcl-2 and Bax^79^. Furthermore, the anti-apoptotic effect of quercetin runs in parallel with the results of Baher et al.^55^, who demonstrated the therapeutic role of quercetin in manganese-induced neurodegeneration in rats suggesting that quercetin inhibited apoptosis via its ability to modulate the expression of Bax and Bcl-2, decreasing the Bax/Bcl-2 ratio.

Histopathological examination confirmed the biochemical findings of CuO-NPs intoxicated rats in the current study and is in harmony with the previous report of Ibrahim et al.^51^ Similarly, another study using silver nanomaterial’s supported our results and showed marked histopathological alterations, including varying degrees of degenerative and vascular changes, in the livers of Swiss albino mice^17^.

In contrast, the CuO-NPs intoxicated rats treated with the combination regimen of DHEA and quercetin showed apparently normal liver architecture without cellular infiltration. Our results are supported with the findings of Arafa et al.^49^ that showed amelioration of CuO-NPs induced hepatotoxicity in rats by the use of quercetin. Also, our results are in agreement with those of Aly et al.^56^ who revealed a relatively normal histological structure of the hippocampus of Al-intoxicated ovariectomized rats treated with DHEA. These findings were attributed to the antioxidant, antiapoptotic and neurotrophic effects of DHEA.

## Conclusion

In conclusion, CuO-NPs exposure caused significant alterations in the antioxidant level leading to oxidative stress, rapid generation of ROS, depletion of GSH, genotoxicity which are represented as plausible key players in CuO-NPs induced liver injury in rats leading to apoptosis. DHEA and quercetin relieve the hazards associated with CuO-NPs administration, whereas the combination of them exhibited a fairly more potent effect. The present work suggests a beneficial effect of DHEA and quercetin or their combination against CuO-NPs induced oxidative stress and hepatotoxicity via blocking the critical control points of apoptosis.
